# Analysis of small nucleolar RNAs reveals unique genetic features in malaria parasites

**DOI:** 10.1186/1471-2164-10-68

**Published:** 2009-02-07

**Authors:** Prakash Chandra Mishra, Anuj Kumar, Amit Sharma

**Affiliations:** 1 Structural and Computational Biology Group, International Centre for Genetic Engineering and Biotechnology Aruna Asaf Ali Road, New Delhi, 110067, India

## Abstract

**Background:**

Ribosome biogenesis is an energy consuming and stringently controlled process that involves hundreds of trans-acting factors. Small nucleolar RNAs (snoRNAs), important components of ribosome biogenesis are non-coding guide RNAs involved in rRNA processing, nucleotide modifications like 2'-O-ribose methylation, pseudouridylation and possibly gene regulation. snoRNAs are ubiquitous and are diverse in their genomic organization, mechanism of transcription and process of maturation. In vertebrates, most snoRNAs are present in introns of protein coding genes and are processed by exonucleolytic cleavage, while in plants they are transcribed as polycistronic transcripts.

**Results:**

This is a comprehensive analysis of malaria parasite snoRNA genes and proteins that have a role in ribosomal biogenesis. Computational and experimental approaches have been used to identify several box C/D snoRNAs from different species of *Plasmodium *and confirm their expression. Our analyses reveal that the gene for endoribonuclease Rnt1 is absent from *Plasmodium falciparum *genome, which indicates the existence of alternative pre-rRNA processing pathways. The structural features of box C/D snoRNAs are highly conserved in *Plasmodium *genus; however, unlike other organisms most parasite snoRNAs are present in single copy. The genomic localization of parasite snoRNAs shows mixed patterns of those observed in plants, yeast and vertebrates. We have localized parasite snoRNAs in untranslated regions (UTR) of mRNAs, and this is an unprecedented and novel genetic feature. Akin to mammalian snoRNAs, those in *Plasmodium *may also behave as mobile genetic elements.

**Conclusion:**

This study provides a comprehensive overview on trans-acting genes involved in ribosome biogenesis and also a genetic insight into malaria parasite snoRNA genes.

## Background

Malaria is a killer disease that is responsible for > 2 million deaths annually [1]. Given the global spread of multidrug-resistant malaria, there is an urgent need for new chemotherapeutic agents. Of the four species of *Plasmodium*, *P. falciparum *(Pf) is the most deadly owing to its ability to cytoadhere and cause complications like cerebral and placental malaria[2]. The genome sequence of *P. falciparum *provided a foundation for studies on this organism, and is being exploited to search for new drug and vaccines candidates. The AT richness of the *P. falciparum *genome poses immense challenges for its thorough computational analysis; in general, a majority of its genes remain not annotated [3,4].

Several antibiotics and drugs target ribosome complexes, the key machinery for translation of mRNAs into polypeptides [5]. Ribosomal biogenesis is an energy consuming and stringently regulated process inside the cell. In eukaryotes, it begins with the transcription of a pre-rRNA molecules, which are modified and processed into smaller mature 18S, 5.8S and 28S rRNAs [6]. Small nucleolar ribonucleoproteins (snoRNPs) play a major role in the maturation of rRNA molecules [7]. snoRNAs are the small metabolically stable non-coding RNAs present in the nucleoli of cells [8]. They play crucial roles in nucleotide modifications, viz methylation and pseudouridylation of various RNAs like rRNA, snRNA, and tRNA. rRNA modification is important for proper functioning of the translation machinery, and deregulation of nucleotide modification can lead to diseases like dyskeratosis congenita [9,10]. This disease is caused by point mutations in the human gene encoding dyskerin, responsible for pseudouridylation. In an another case, snoRNA HBII-52 is involved in the regulation of mRNA processing [11].

According to the conserved sequence motif and structural features, snoRNA [except the RNA component of RNase MRP (mitochondrial RNA processing)] can be classified into two groups: box C/D and box H/ACA snoRNAs [12]. Box C/D and box H/ACA snoRNAs guide rRNA 2'-O-ribose methylation and pseudouridylation respectively by base pairing with the substrate RNA molecules [13]. Box C/D snoRNAs contain two conserved sequence motifs, box C (5'-RUGAUGA-3'; R-Purine) and box D (5'-CUGA-3') which become proximal to each other due to short stems which constitute the structural core motif of the snoRNA. Commonly, box C/D and H/ACA snoRNAs are about 65–100 and 120–160 bases in size respectively [14]. In vertebrates, snoRNAs are predominantly located in introns of ribosomal proteins and housekeeping genes, whereas in yeast, most of them are transcribed from independent promoters [15,16]. Intron-encoded snoRNA genes can follow different pathways for maturation: splicing dependent or independent [17].

In most eukaryotes, the copy number of rRNA genes is high and they are present as tandem repeats. However, *Plasmodium *genome is unique amongst eukaryotes where rRNA genes have very few copies, and they are present on different chromosomes [3,18]. Moreover, two distinct types of developmentally regulated, cytoplasmic rRNA transcripts have been detected in the parasite[19]. One type of transcript is predominant in the asexual stages of the parasite life cycle, and the other in the sexual stages. The ratio of these two types of transcripts changes dramatically during the developmental cycle of the parasite, but neither of them disappears completely at any stage of the life cycle[19]. Both of them are distinct in length and also differ in sequence in some non-conserved regions. Since the highly conserved genomic organization of rRNA genes amongst eukaryotes becomes different in *Plasmodium*, all aspects related to ribosome biogenesis in *Plasmodium *call for special interest. A better understanding of ribosome structure and its biogenesis pathways may help in the development of anti-malarial drugs. snoRNAs and other trans-acting proteins comprise an important component of ribosomal biogenesis, making their identification and analysis of their genetic organization and evolutionary linkages important for understanding their role in *Plasmodium*.

Genes corresponding to snoRNAs have been identified in rice, *Drosophila*, yeast and various other organisms [20-25]. Recent studies reveal that snoRNAs are a new class of non-autonomous mobile genetic elements that traverse using RNA intermediates[26,27]. In the present work, we provide a comprehensive analysis of the snoRNA genes present in malaria parasites. Our study reveals the following key features of malaria parasite snoRNAs: (1) structural features of box C/D snoRNA are highly conserved in *Plasmodium *genus, (2) unlike other organisms, most snoRNAs in malaria parasites are present as a single copy, (3) genomic localization patterns are mixture of those observed in plants, yeast and vertebrates; parasite snoRNAs are present in clusters and introns of a gene (4) we have found snoRNAs in 3'UTR of an mRNA, a feature not reported in any organism till date, (5) and finally, we propose that as in mammals, the parasite snoRNAs may behave as mobile genetic elements.

## Results

### Proteins involved in ribosomal biogenesis

Considering the unusual gene structure and developmentally regulated transcription of ribosomal RNA in *Plasmodium*, we have tried to study the process of ribosomal biogenesis in this genus. Amongst all the eukaryotes, ribosomal biogenesis is most studied in yeast, leading to identification of various genes important for processing and maturation of pre-rRNA. We have used sequences of proteins involved in yeast ribosomal biogenesis pathways to find their homologues in Pf and tried to delineate pathways of ribosomal biogenesis in *Plasmodium*. We have searched for all the ribosomal proteins in Pf genome and also the trans-acting factors that have a role in ribosomal biogenesis (results listed in additional file 1).

*Plasmodium falciparum *genome contains genes corresponding to all ribosomal proteins that are present in small and large subunits of eukaryotic ribosome, except RPL29. These ribosomal proteins share high sequence identity with their counterparts in yeast and human. Ribosomal proteins are unlike other Plasmodia proteins that are generally larger than their homologues in other eukaryotes.

As soon as RNA polymerase transcribes pre-rRNA, the transcript is modified, mainly by pseudouridylation and 2'-O-ribose-methylation, at specific sites selected by snoRNP complex[28]. This is followed by cleavage in 3' external transcribed spacer (ETS) by an endoribonuclease Rnt1p, a homologue of bacterial RNase III. Box C/D snoRNP complex, responsible for 2'-O-ribose-methylation is constituted of a small RNA molecule (box C/D snoRNA) and various proteins (fibrillarin, Nop56, Nop58 and Snu13). Similarly, box H/ACA snoRNP complex responsible for psuedouridylation is comprised of a RNA molecule (box H/ACA RNA) and proteins (Cbf5, Nhp2, Nop10 and Gar1). Through our analysis we could find homologues of each protein present in the snoRNP complexes, but the gene corresponding to Rnt1p enzyme was absent from *Plasmodium *genome, when searched using PSI-BLAST (for eight iterations).

Exosome is a multi-protein complex of 3'-5' exoribonuclease which is responsible for maturation and processing of pre-rRNA and many other RNA like pre-mRNA and small RNAs[29]. Exosome is composed of 9–11 subunit proteins, of which six (Rrp41, Rrp42, Rrp43, Rrp45, Rrp46, and Mtr3) have sequence identity with the E. coli RNase PH domain. Genes corresponding to Rrp4, Rrp6, Rrp40, Rrp42, Rrp44, Rrp45 and Csl4 could be identified in the *Plasmodium *genome. However, homologues of Rrp43 and Mtr3 could not be found in Pf genome, whereas Rrp41 and Rrp46 shared homology with a single protein PF14_0256.

### Identification of putative box C/D snoRNA

An analysis of methylation sites from various organisms reveals that rRNA 2'-O-methylation sites are highly conserved[30]. We therefore used known yeast and human methylation sites to find putative methylation sites in Pf ribosomal RNAs by aligning its 18S and 28S rRNA (both asexual and sexual stages) sequences with those from yeast and human. Each of the mapped methylation sites in asexual and sexual stage rRNA of *Plasmodium falciparum *were observed to lie in conserved regions. For further studies we considered only the asexually expressed rRNA sequences. The program SNOSCAN was used to predict snoRNA genes in the Pf genome [20]. More than 100 box C/D snoRNA genes were predicted for different methylation sites in the small (SSU) and large subunits (LSU) of ribosomal rRNAs. To eliminate false positive snoRNA genes, we used the following strategy – methylation sites generated from the above-mentioned alignment were matched with the sites predicted for putative snoRNA (identified using SNOSCAN) to generate a subset of relevant snoRNA genes (for matched methylation sites). Interestingly, none of the snoRNA genes present in this subset localized to the protein-coding regions. They were present either in introns or in intergenic regions, and had canonical box C (UGAUGA) and box D (CUGA) motifs. Therefore, after removing all those predicted snoRNAs, which were either localized in the protein coding regions or did not have canonical box C and D motifs, 16 snoRNA were left.

**Figure 1:**
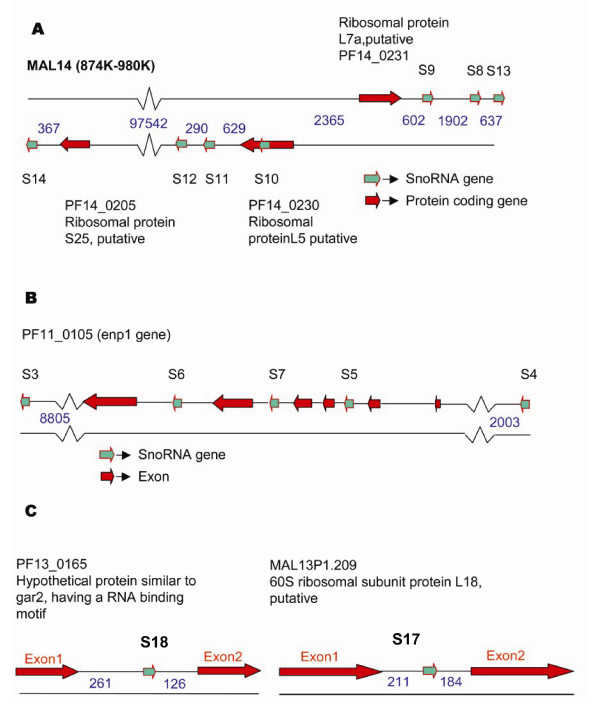
**Localization of snoRNA genes in *P. falciparum***. A) snoRNAs present on chromosome 14 between regions 874 k–980 k. B) snoRNA genes present in introns of enp1 gene and it's flanking regions. C) Two snoRNA genes localized in introns of two ribosomal proteins on chromosome 13. Numbers in blue color are the distances given in nucleotide bases.

We also extracted 1000 nucleotides upstream and downstream of each of these 16 snoRNA gene and rescanned them through SNOSCAN program with lower cutoffs. Different introns of a gene harbouring a snoRNA were also searched for other low scoring box C/D snoRNA genes. This helped us identify two additional snoRNA genes, PFS5 and PFS10 in the introns of PF11_0105 and PF14_0230 respectively. Table 1 summarizes the 18 snoRNAs of Pf, and sequences of these are given in Table 2. A schematic representation of some of the snoRNA genes in context of the genome is shown in Figure 1. We have named the genes with the initial two characters representing the species and the following characters specify snoRNA number. For example PFS1 represents *P. falciparum *snoRNA1 and PVS6 refers to *P. vivax *snoRNA 6.

**Table 1:** Summary of 18 box C/D snoRNA genes in *P. falciparum*.

**snoRNA**	**Chr**	**Methylation Site**	**Start**	**End**	**Location**	**Homology to yeast**
**PFS1**	3	18S Tm1370, 28S Gm1058 Gm3308	398237	398159	Non coding, intergenic region	SSU Um1269

**PFS2**	8	18S A1129 28S Am3307	1162400	1162306	Intron of PF08_0019	LSU Am2946

**PFS3**	11	18S Cm1936, 28S Cm2490	378872	378789	Non coding, intergenic region	SSU Cm 1639

**PFS4**	11	18S Am28	392950	392870	Non-coding, intergenic region	SSU Am28

**PFS5**	11	28S Gm2960	389451	389379	Intron of PF11_0105	LSU Gm2619

**PFS6**	11	28S Am1264	388122	388057	Intron of PF11_0105	LSU Am1133

**PFS7**	11	28S Cm3176	388761	388683	Intron of PF11_0105	LSU Gm2815

**PFS8**	14	18S Gm1798, 28S Gm926	981730	981811	Downstream of RPL7a (PF14_0231)	LSU Gm805

**PFS9**	14	28S Cm3240	979909	979977	3' UTR of RPL7a (PF14_0231)	LSU Cm4426 (Human)

**PFS10**	14	18S Gm1674	974214	974149	Intron of PF14_0230	SSU Gm1428

**PFS11**	14	18S Am442	973365	973290	Downstream of RPL5 Family (PF14_0230)	SSU Am 436

**PFS12**	14	18S Am1043	972999	972920	Downstream of RPL5 Family (PF14_0230)	SSU Am974

**PFS13**	14	28S Am728	982297	982367	Downstream of RPL7a (PF14_0231)	LSU Am649

**PFS14**	14	28S Am2551	875464	875318	Downstream of RPS25 (PF14_0205)	LSU Am2256

**PFS15**	14	28S Cm2632	92872	92794	Intron of PF14_0027	LSU Cm2337

**PFS16**	14	28S Cm2632	93091	93013	Intron of PF14_0027	LSU Cm2337

**PFS17**	13	28S Cm3320	1637301	1637376	Intron of MAL13P1.209 60S ribosomal subunit protein L18, putative	LSU Cm2959

**PFS18**	13	28S Cm1589	1295143	1295218	Intron of PF13_0165	LSU Cm1437

Chr means chromosome number, LSU and SSU – large and small subunit of ribosome respectively

**Table 2:** Sequences of box C/D snoRNA genes in *P. falciparum*.

**snoRNA**	**Sequence**
**PFS1**	CAATA**TGATGA**TAAACATTACCCAGCTCAT*CTGA*AGTATATAACCATGAAGATATTTTTT CATGCATCACAAT**CTGA**TT

**PFS2**	TTATA**TGATGA**CAAGTGACTATCCCAGCTCACT*CTGA*TTTTTATTTTTAAAATGAAGAGA AAATAGCTCATATTATTTTATTAATTTTT**CTGA**TA

**PFS3**	TAAAA**TGATGA**ATAACTTTTGAGCGATGGGCGGA*CTGA*AAAAAGTGAGAGAACTTTTATT TGTAGAAAATCGCATAAT**CTGA**TA

**PFS4**	TTCTG**TGATGA**TTTTGTATAAATTATTTGACAAGCATATGT*CTGA*TAAAAATTTATT***TGATGA***AATTTTTGAAAA**CTGA**GA

**PFS5**	TTATA**TGATGA**TTAGTCTTGT*CTGA*ATATTTAATTTTAAAAAATGAAGACAATAGTACTG CCCCAAA**CTGA**TT

**PFS6**	TGAAA**TGATGA**AACAAAACAGTTCTGCTTCTGAATTTATTT***TGATGA***TAACTATGCCCAA **CTGA**TC

**PFS7**	TTAAA**TGATGA**AAACGTACGCTTGGCATCTGAATAATTTATTTGAAGATAAATTTTTAAT CAGTTATCCCTAT**CTGA**TA

**PFS8**	AAAAA**TGATGA**CAACCTTTTCATAATATAAAGCCTTTCGGGTCTGAAGAGCATTA*TGATGA*TAAAAAAAAAAAAAA**CTGA**TT

**PFS9**	TATAA**TGATGA**AAACTTCAAGGAAGTGCCGT*CCGA*ATATTTATGTTGACGATAATTAATT AAT**CTGA**AC

**PFS10**	TTTAA**TGATGA**GAAAAACAGACCTGAT*TTGA*AAAATAATTTGAGAATAATTATAACGCTC **CTGA**AA

**PFS11**	TTTAA**TGATGA**CTGAATAAATAATATGTGGGTAATTTACGTCTGAAAATTAT***TGATGA***TT ATTATAGTAT**CTGA**AA

**PFS12**	GTATA**TGATGA**ATAAAAAAAAATTATTTAACTTTCGTTCTTCTGCATCTTTTGAAGTGAA GATAAATTTTATAT**CTGA**TA

**PFS13**	TTTAA**TGATGA**TATGAAGAACTTGGTCTGTGTTACTGAAATAATATAGAGATGAAAAAAA AACGA**CTGA**AA

**PFS14**	TAAAG**TGATGA**TAAAAAAAAAATATAAAAAAAGGTGATGCGGAACTTAAAAAAAATGTAA TAAAAGATTTCTTCTTATATTCATTTTATTTTATTTCTGCCAAAAAAGAAAAATGAGCCTTAT ATAGAAGTCATAGTTACT**CTGA**TT

**PFS15**	TTTTA**TGATGA**TACAATTCCAAAAATGCAAGTAGGGAC*ATGA*GAATATTATAAATATGTT CGTCTTCTATTAT**CTGA**AA

**PFS16**	TATAA**TGATGA**TAAATAAAAATATGCAAGTAGGGAC*ATGA*GAAAACTTTTATTATGTTCG ATATTTTTACCTT**CTGA**TT

**PFS17**	TTACA**TGATGA**ATAAGCTTCTACGAATCACGACGGTCTTCTGACATTATCAATGGAGATG GTAGAACGTT**CTGA**TC

**PFS18**	TTTTA**TGATGA**AAATAAAAAAGAAAAG*CTGA*TAAAAAGT*TGATGA*TTTTTATGCTATTTC ACCAAGATCT**CTGA**AA

Box C and D are depicted in bold. Box C' and D' are labelled in italics

### Homologues of Pf snoRNA genes from other *Plasmodium *species

Orthologous loci of other *Plasmodium *species (*P. chabaudi*, *P. yoelii*, *P. vivax*, *P. knowlesi *and *P. gallinaceum*) were searched for snoRNA genes, as outlined in additional file 2. Of the 18 predicted box C/D snoRNA genes from *P. falciparum*, most have potential orthologs in other *Plasmodium *species indicating the highly conserved nature of snoRNAs in this genus. However, homologues of PFS16 could not be found in any other species of *Plasmodium*. PFS16 is a paralog of PFS15 since both guide the same methylation site, suggesting that this may be a recent case of gene duplication. Phylogenetic analysis of homologous snoRNAs revealed that *P. vivax *most likely diverged from *P. falciparum *very early. The data indicate that *P. knowlesi *is evolutionarily closer to *P. vivax*, whereas *P. falciparum *seems more related to *P. gallinaceum *(Data not shown). These evolutionary pattern retained phylogenetic deductions made by analysis of other conserved genes like rRNA genes [31].

### snoRNA expression analysis by northern hybridization and RT-PCR

**Figure 2:**
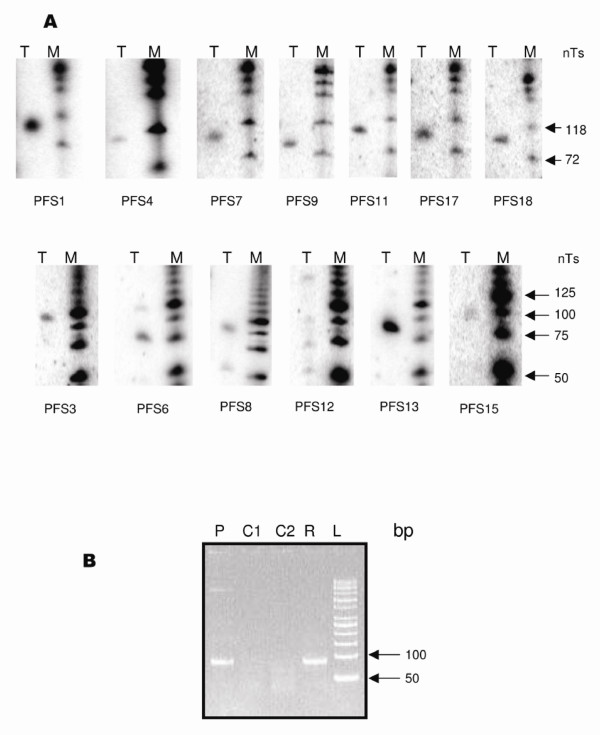
**Expression of snoRNA in *P. falciparum***. A) Northern hybridization of snoRNAs in *P. falciparum *3D7. Total RNA was size fractionated on 10% urea-polyacrylamide gels. Blots were probed using labelled DNA primers. T and M stands for total RNA and molecular weight marker respectively. B) Reverse-transcriptase PCR assay for PFS14 using forward primer PFS14_F and reverse primer PFS14. P is the positive control containing genomic DNA as template, C1 is a negative control that lacks a template, C2 is a negative control containing cDNA generated using DNA polymerase and R is the reaction that contains cDNA generated using reverse transcriptase enzyme. L is the DNA ladder.

Parasite RNA was isolated from *in vitro *cultured erythrocytic stages of Pf parasites. Expression of each of the predicted Pf snoRNA was tested using two independent techniques – northern hybridization and reverse transcriptase PCR assays. For the latter, total RNA was used, whereas for northern hybrization we used RNA enriched in small RNA. Out of the 18 predicted snoRNA genes, expression could be confirmed for 14; 13 using northern hybridization and one using reverse-transcriptase PCR assay (Fig 2). Multiple bands could be observed in northern blots of PFS6, PFS8 and PFS12, most likely owing to their processing from longer pre-snoRNA transcripts. PFS12 northern blot has two major bands, one corresponding to the size of the snoRNA and other probably to pre-snoRNA. The other low intensity band may be due to processing intermediates and degradation products. No experimental evidence for the expression of PFS2 and PFS16 was found, which may be either due to low number of detectable transcripts or lack of transcription of these genes in the erythrocytic stages of parasites. We were also able to find sequences of many snoRNAs in the expressed sequence tags (EST) database of PlasmoDB (Table 3), an observation that validates snoRNA expression in other species of *Plasmodium*.

**Table 3:** Results of EST database search

**snoRNA**	**PF**	**PV**	**PY**	**PB**
**S3**	N.A.	CV644718	N.A.	N.A.

**S4**	N.A.	N.A.	N.A.	BB976413

**S6**	AU086774	CX018826	N.A.	N.A.

**S8**	N.A.	N.A.	N.A.	BB979125
				BB971849
				BB973622
				BB980710

**S9**	N.A.	CV644537	N.A.	BB980593

**S11**	N.A.	CX022489	N.A.	N.A.
		CX020332		
		CV645856		
		CV645000		
		CV641405		
		CV633925		
		CV633826		

**S12**	N.A.	CV645856	CF468858	N.A.
		CV645000		
		CV641405		
		CV633925		
		CV633826		
		CX022489		

**S13**	N.A.	CX022449	N.A.	N.A.
		CV648424		

**S14**	BU494865	CB065559	BM160472	N.A.

**S17**	N.A.	N.A.	BM162361	BB977848
			BM169056	

EST ID number from PlasmoDB (NA – no sequence was found)

### Structural features of *P. falciparum *box C/D snoRNAs

The two conserved motifs, box C and D are present in all snoRNA genes, and are immediately followed by a 4–10 bases inverse repeat, which forms the terminal stem structure that brings the two boxes in close proximity. This is an important feature required for the stability and function of snoRNPs [8]. Some snoRNAs also contain two antisense motifs (near D and D' box) complementary to different target sequences. We were able to identify 18 box C/D snoRNA genes exhibiting all the canonical structural features including box C, box D, terminal stem and at least one region (8–15 bases) complementary to the rRNA. Some deviations were observed in the D' sequence of some snoRNA genes (for e.g. ATGA in PFS15 and PFS16, CCGA in PFS9, TTGA in PFS10). In the case of PFS10, the CTGA motif is a part of the antisense element (region complimentary to rRNA), while TTGA forms the D' box. A total of four (out of 18) snoRNAs have two antisense regions, which may be responsible for methylation at two different sites instead of one. PFS2 shows a unique feature of having two antisense regions in tandem, one of which matches with the antisense region of PFS1. Five (out of 18) snoRNAs have a box C' motif (PFS4, PFS6, PFS8, PFS11 and PFS18). For all snoRNAs present in introns (except PFS16), the distance between splice sites and the snoRNA ends were greater than hundred bases, which is required for splicing dependent maturation of snoRNA [17].

Contrary to the general observation that Pf genes are longer than their counterparts in other organisms, size of snoRNA identified in this study falls in the generally observed range of 70–90 bases, with an exception of PFS14, which is 147 nucleotides long [32]. Interestingly, a homologue of PFS14 in *P. vivax *is only 93 nucleotides in length, as a polyA repeat present in other species is absent from *P. vivax*. Additionally, a conserved polyA followed by TA-repeats was observed downstream of many snoRNA genes. PolyA repeat is a common feature observed downstream of retroposons.

### The genomic organization of box C/D snoRNA in malaria parasites

**Figure 3:**
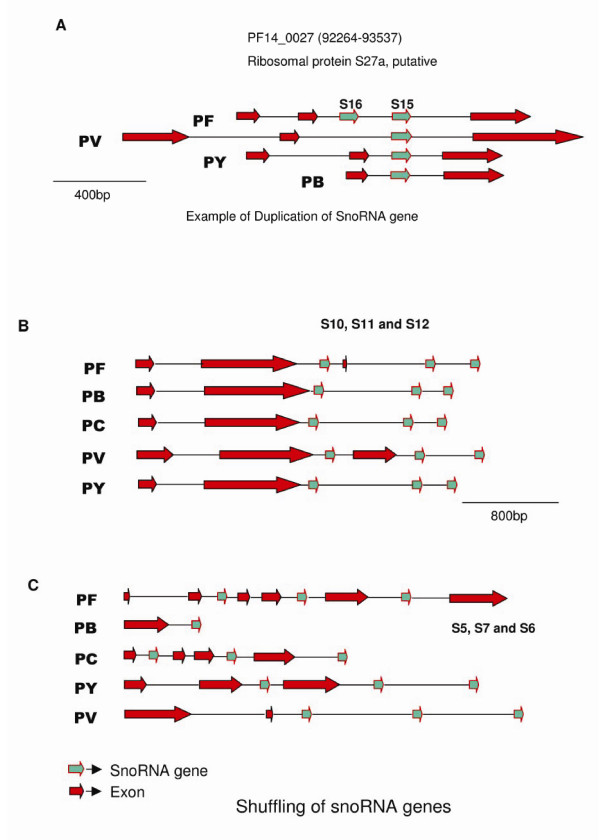
**Localization of homologous snoRNA in different species**. A) Example of snoRNA duplication. S16, a paralog of S15 is absent in other species. B) snoRNAs S10, S11 and S12 are present differently in other species. Notably, order of the gene is same in every species. C) Localization of S5, S6 and S7 in different species of *Plasmodium*. Order of the genes remains same in every species.

**Figure 4:**
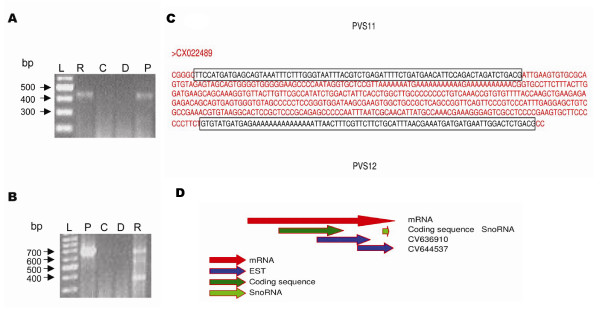
**Localization of snoRNA**. Reverse transcriptase PCR assays showing A) cluster of PFS11 and PFS12 (using forward primer against PFS11 and reverse against PFS12; the primers sequences are ATGATGACTGAATAAATAATATG and TCAGATATAAAATTTATCTTCAC respectively) and B) Localization of PFS9 in 3' UTR of protein coding gene; P is the positive control containing genomic DNA as template, D is a negative control that lacks a template, C is a negative control containing cDNA generated using DNA polymerase and R is the reaction which contains cDNA generated using reverse transcriptase enzyme; L is the 100 bp ladder from Fermentas. C) Sequence of an EST from *P. vivax*. PVS11 and PVS12 are shown within a box in the figure. D) Localization of PVS9 in the mRNA, Sequence of two overlapping EST are identical with snoRNA at its 3' end and protein coding sequence at 5' region.

Genomic organization of snoRNA genes varies from one species to another. Nine out of 18 snoRNA genes (PFS8–PFS16) in Pf are present on chromosome 14, and the rest of them are distributed on chromosomes 3, 8, 11 and 13 (Table 1). Nine out of 18 snoRNAs in Pf are present in introns of protein-coding genes. Another 6 are localized downstream of gene encoding ribosomal proteins. PFS2 is located in an intron of the gene for guanine nucleotide binding protein. This protein is a mediator for many cellular processes, including signal transduction, protein transport, growth regulation and polypeptide chain regulation [33]. PF11_0105, a homologue of Enp1 protein, harbors snoRNAs PFS5, PFS6 and PFS7 (Fig 3). Enp1 protein has a role in early processing and maturation of ribosomal RNA [34]. Two of the snoRNAs indentified, PFS15 and PFS16 are in the same intron of ribosomal protein S27a, a feature unlike vertebrates but similar to plants, which have a cluster of snoRNA genes in the same intron [15,35]. Three snoRNAs PFS8, PFS9 and PFS13 are present downstream of PF14_0231, a ribosomal protein of L7a family, while PFS11 and PFS12 are present downstream of PF14_0230 that belongs to the L5 family. The last intron of PF14_0230 gene carries a snoRNA PFS10, which shifts its locus downstream of the stop codon for the same gene in other *Plasmodium *species, except in *P. vivax *(Fig 3B). PFS14 is present downstream of protein PF14_0205 which is homologous to a ribosomal protein S25. The intergenic distance between PFS11 and PFS12 is just 290 bp, which is reminiscent of two genes transcribed as a single transcript and further processed into mature snoRNA. In order to test the same, we performed a reverse transcriptase PCR assay using specific forward primer against PFS11 and reverse primer against PFS12. Our results show (Fig 4) that these snoRNAs are present in a cluster and are transcribed as a longer RNA transcript together from a single promoter. They are subsequently processed further into two mature snoRNAs, PFS11 and PFS12. Similarly, PVS11 and PVS12 in *P. vivax *are also coded as a polycistronic gene. Both these snoRNAs are localized in the sequence of an expressed sequence tag (EST) CX022489 of *P. vivax *(Fig 4).

### The 3' UTR harbours snoRNA genes

Many of the identified snoRNAs from Pf (like PFS9, PFS11 and PFS14) lie just 602, 629 and 367 bases downstream of the stop codon of genes PF14_0231, PF14_0230 and PF14_0205 respectively, and are likely to be contained within their 3' UTR. We performed reverse transcriptase PCR assays using specific forward primer against the 3' end of their protein coding region and reverse primer specific for the snoRNA gene. Our results show (Fig 4) that PFS9 is located in the 3' UTR of the ribosomal protein L7a. In the case of PFS11, we did not observe any amplified product, which implies that it does not lie in the UTR. For PFS14, a conclusive result could not be obtained, as the amplified product was smaller than the expected size (data not shown). PVS9, the homologue of PFS9 in *P. vivax *was also localized to the 3' UTR of the ribosomal protein L7a (Fig 4). PVS9 is present in the sequence of an EST CV644537, whose sequence overlaps with the 3' end of another EST CV636910. Sequence of the 5' end of CV636910 was identical to the coding region of ribosomal protein L7a in *P. vivax*. So, using sequences of these two overlapping ESTs, we calculated that the length of 3' UTR of ribosomal protein L7a gene should be at least 681 bases. Since the distance between stop codon of this gene and PVS9 is just 560 bases, the snoRNA should be present in the 3' UTR of the ribosomal protein L7a gene. This is a novel gene organization not reported in any other organism till date.

### Identical copies of PKS11 with polyA tail

**Figure 5:**
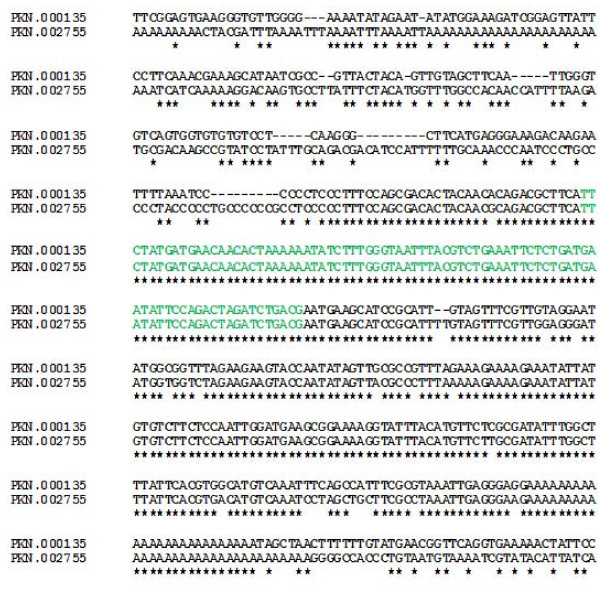
**Retrotransposon**. Sequence alignment of two loci containing snoRNA PKS11, snoRNA sequence is highlighted in green.

Two identical copies of a PKS11, which is a homolog of PFS11, are present at different loci in the *P. knowlesi *genome (one at PKN.000135 and another PKN.002755). snoRNA gene on PKN.002755 has polyA repeats at 5' and 3' ends whereas PKN.000135 has poly A repeats at 3' end only (Fig 5). Other species of *Plasmodium *have only one snoRNA at similar loci as of PKN.002755.

## Discussion

The aim of this work was to identify various genes involved in ribosomal biogenesis pathway of malaria parasite. Various proteins and RNA molecules like RNA helicases, U3 snoRNA, RNA MRP have been identified and reported earlier in Pf [36,37]. The structures and sequences of 5' ETS of the different stage specific pre-rRNA are also known from a previous study, but no information is available for 3' ETS[18]. The present study highlights the differences in the pathway, where gene for a particular function (either protein or RNA) is missing in the Pf genome. Absence of any factor would indicate deviation from the known pathway and would call for identification of alternative mechanisms present in malaria parasites.

In spite of the differences in the gene structure and transcription pattern of rRNA genes in *Plasmodium*, the protein components of ribosome do not show much difference. We could identify homologues for each of the proteins present in large and small subunit of the ribosome, except RPL29. The sequence identity with yeast and human homologues for most of the ribosomal proteins were higher than 60%, which indicate conservation of protein structure and function.

Most of the trans-acting factors responsible for ribosomal biogenesis are present in the parasite genome. Proteins present in the various complexes like box C/D snoRNP, box H/ACA snoRNP, U3 snoRNP and exosome have their homologues in the parasite genome. We were unable to find a homologue for the endoribonuclease Rnt1p, responsible for the cleavage in 3' ETS of pre-rRNA. This step of ribosomal biogenesis in malaria parasite seems to differ from yeast and the protein responsible for this function in Pf may be a drug target. Further study is required to identify the sequences of 3' ETS of pre-rRNA and various proteins involved in the alternative pathway for its processing.

Chakrabarti *et al *have reported the existence of 2'-O-methylation and snoRNAs in malaria parasites[37]. They have identified non coding RNAs using GC content, RNA folding potential and sequence conservations. We have predicted box C/D snoRNA genes of *P. falciparum *using a different computational method based on hidden markov model (HMM), which has enabled us to identify even those snoRNA which are present in only one species. Identification of species specific snoRNA helps to understand the mechanism of snoRNA evolution. Out of the 18 snoRNA genes in this study, 16 were reported by Chakrabarti *et al*. Additionally we have identified some new snoRNA genes not listed in the previous report. These are: PFS16, which guides methylation on a site identical to that for PFS15; and PFS14, whose size differs amongst species due to a small AA rich insertion in the gene. Additionally, we also report the presence of two identical copies of PKS11 in *P. knowlesi *genome. Identification of gene duplication in the genome shed new light on the mechanism of evolution of snoRNAs in *Plasmodium *genus.

**Figure 6:**
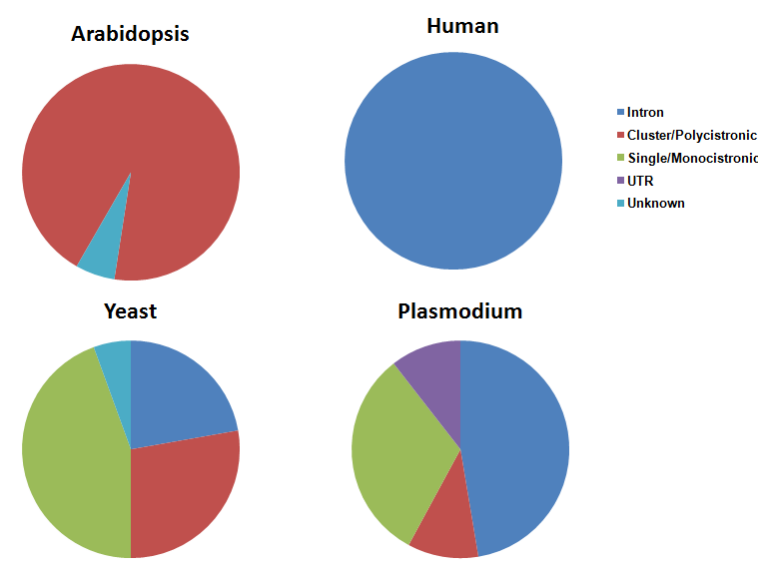
**Genomic organization of snoRNA**. Graphical representations of localization pattern of the identified 18 snoRNAs in *P. falciparum *and the snoRNAs having similar methylation sites in human, Arabidopsis and yeast. In Arabidopsis, localization pattern of 16 snoRNAs are represented.

We have confirmed the expression of snoRNA genes using northern hybridization and reverse transcriptase PCR assays. We have also identified the orthologs of these genes in other *Plasmodium *species. A comparative study of these snoRNAs has revealed features unique to malaria parasite. Most of the snoRNAs in vertebrates are localized to introns of protein coding genes [15,38]. In yeast, most snoRNAs are transcribed from their independent promoters [39], barring a few intron-encoded genes. In the case of *Plasmodium*, we found a mixture of localization patterns observed in yeast, vertebrates and plants (Fig 6). Table 4 summarizes the comparative study of these snoRNAs, all of which have similar target sites in Pf, yeast, humans and Arabidopsis. All the 18 human snoRNAs are located in introns as compared to only nine in case of Pf and four for yeast. Eight of the snoRNAs in yeast are monocistronic and five exist as polycistronic genes. In the case of Arabidopsis, all these are present in cluster and are transcribed as polycistronic genes, whereas, in Pf two gene clusters were observed, one in the case of PFS11 and PFS12 and another in the case of PFS15 and PFS16 (Fig 4). In both vertebrates and yeast, one intron harbours single snoRNA gene but in plants, there are reports of clustered snoRNA genes present in a single intron [35,40]. *Plasmodium falciparum *has a cluster of two snoRNAs, viz PFS15 and PFS16, which are present in the same intron of PF 14_0027 (Fig 3).

**Table 4:** Localization of homologous snoRNA genes

**snoRNA**	**Yeast**	**Human**	**Arabidopsis**
**PFS1 (S)**	snR55 (M)	U33 (I)	AtsnoR34 (C)

**PFS2 (I)**	snR71 (M)	U29 (I)	AtsnoR69 (C)

**PFS3 (S)**	snR70 (P)	U43 (I)	AtU43 (C)

**PFS4 (S)**	snR74 (P)	U27 (I)	AtU27 (C)

**PFS5 (I)**	snR67 (P)	U31 (I)	AtsnR31 (C)

**PFS6 (I)**	snR61 (P)	U38 (I)	AtU38 (C)

**PFS7 (I)**	snR38 (I)	snR38 (I)	AtsnoR38 (C)

**PFS8 (S)**	snR39b (M)	snR39b (I)	AtsnR39BY (C)

**PFS9 (UTR)**	N.A.	U49 (I)	AtU49 (C)

**PFS10 (I)**	snR56 (M)	U25 (I)	AtsnoR19 (C)

**PFS11 (C)**	snR87 (M)	U16 (I)	AtU15 (C)

**PFS12 (C)**	snR54 (I)	U59 (I)	AtsnoR59 (C)

**PFS13 (S)**	U18 (I)	U18 (I)	AtU18 (C)

**PFS14 (S)**	snR63 (M)	U46 (I)	N.A.

**PFS15 (I, C)**	snR64 (M)	U74 (I)	AtsnoR44 (C)

**PFS16 (I, C)**	snR64 (M)	U74 (I)	AtsnoR44 (C)

**PFS17 (I)**	snR73 (P)	U35 (I)	AtU35 (C)

**PFS18 (I)**	U24 (I)	U24 (I)	AtU24 (U)

Genomic localization of homologous snoRNA genes of yeast, human and Arabidopsis, which guide methylation of similar sites as in *Plasmodium*. Localization information is given in the adjacent right hand side brackets. UTR, P, C, M, S, U and I stand for untranslated region, Polycistronic, Clustered, Monocistronic, single C/D box snoRNA in intergenic region, Unknown and Intron-encoded respectively.

Trypanosomatids are unicellular parasitic protozoa which are the causative agents of several infamous parasitic diseases, such as African trypanosomiasis, caused by *Trypanosoma brucei*; Chagas' disease, caused by *Trypanosoma cruzi*; and leishmaniasis, caused by *Leishmania *species and have a dual host like malaria parasites. Most snoRNAs in these organisms are clustered in reiterated repeats that carry a mixed population of C/D and H/ACA-like RNAs[41]. Prediction of the modifications guided by these RNAs and using partial mapping data, 84 2'-*O*-methyls (Nms) and 32 pseudouridines were identified on rRNAs, suggesting a high occurrence of Nms as compared to pseudouridines on rRNA. Occurrence of a mixed population of box C/D and H/ACA snoRNAs and a higher number of Nms than psuedouridylation is in line with findings in *Plasmodium *as reported by Chakrabarti *et al*[37].

The functional and evolutionary significance of UTRs of eukaryotic transcripts remains unclear. There are reports of introns in the UTRs of RNA transcripts but their functional significance is unknown[42]. In this work, we show that snoRNA PFS9 is contained within the 3' UTR of the RNA transcript of ribosomal protein L7a using RT-PCR. Identification of polyA site of the mRNA using 3' RACE would be another method to prove this. The homolog of PFS9, PVS9 in *P. vivax *is also located in the 3' UTR of the ribosomal protein L7a gene. Localization of snoRNAs in 3' UTR is a novel organization not reported in any other organism till date.

PFS10 is located in an intron of a ribosomal protein PF14_0230 but its homologues in *P. chabaudi*, *P. berghei *and *P. yoelii *are present downstream of the last exon. The three snoRNAs PFS5, PFS6 and PFS7 are present in introns of Enp1 gene of Pf but are localized differently in different species (Fig 3B and 3C). From these observations, it seems likely that human and malaria parasite snoRNAs prefer to localize in introns rather than in intergenic regions. Harbouring snoRNA genes in introns and UTRs of a constitutively expressed gene may be a more efficient and coordinated method for transcription. In case of *P. vivax*, the mRNA of Enp1 gene does not code for the full protein. It is probable that the mRNA harbouring snoRNA genes has lost its translational capability, which is reported in many cases [43].

An interesting observation shows that PFS1 and PFS2 are evolutionarily linked and may have evolved from a common ancestor. Both PFS1 and PFS2 have an identical antisense region. PFS1 has two regions complementary to rRNA, one for 18S Tm1370 and another for 28S Gm3308, whereas PFS2 has for 18S A1129 and 28S Am3307. Regions for 28S – 3307 and 3308 are similar, except for deletion of a cytidine from PFS1 antisense region, leading to change in target site.

Studies on mammalian snoRNA genes have revealed that they are a new class of mobile genetic elements [26,27]. It has been proposed that retroposition followed by genetic drift is a mechanism that can increase snoRNA diversity during vertebrate evolution to eventually acquire new RNA-modification functions. In this study, our results imply that this mechanism may hold true in *Plasmodium *also. In the first case, two identical copies of a homologue of PFS11 are present in the *P. knowlesi *genome, whereas only a single copy is present in other species. Sequence alignment of these two loci with flanking regions suggests a 'copy-paste mechanism', as observed in case of retrotransposons (Fig 5). We propose that snoRNA duplication may be due to their behaviour as snoRTs because of two reasons 1) both the copies are 100% identical with few bases of overhangs and one of them had a poly A at the 5' end of the sequence (present on PKN.002755), whereas the other copy doesn't have it; presence of polyA tail is an important feature of transposons that traverse using RNA intermediates 2) Only *P. knowlesi *has two copies whereas other species of *Plasmodium *have only one copy. The parental copy may be the snoRNA present on PKN.000135 which may have been lost during evolution in other species. Since this is the only identified cases of snoRT in *Plasmodium*, our hypothesis cannot be conclusively verified. Similar studies on H/ACA snoRNAs may help to draw a final conclusion. In another case, PFS15 is conserved in *Plasmodium *species but its paralog – PFS16, is absent from another species, indicative of gene duplication.

## Conclusion

We have identified snoRNA genes in malaria parasite and have done a comprehensive analysis of their structural features and genomic organisation, which has helped us to understand the mechanism of evolution of snoRNA genes. Like rRNA genes, these are present in low copy numbers and their patterns of gene organization are a mixture of those observed in other organisms. A feature unique to *Plasmodium *is the localization of snoRNA genes in 3' UTR of the ribosomal protein genes. As in mammals, snoRNAs may behave as retrotransposons in *Plasmodium *and may be one of the mechanisms of snoRNA evolution. The gene for the endoribonuclease Rnt1 is absent from malaria parasites genome, which indicates the existence of alternative pre-rRNA processing pathways as compared with the one in yeast.

## Methods

### Identification of *Plasmodium *snoRNAs

The genome sequence of *Plasmodium falciparum *3D7 was downloaded from PlasmoDB [44]. Ribosomal RNA gene loci were deleted from the genome sequence to avoid false positive hits, which was then used for searching potential box C/D snoRNA genes using snoRNA search program SNOSCAN [20]. SNOSCAN is based on a greedy search algorithm. It sequentially identifies six features in the gene: box C, box D, a region of sequence complementary to ribosomal RNA, box D' if the rRNA complementary region is not directly adjacent to box D, the predicted methylation site within the rRNA based on the complementary region and the terminal stem base pairings, if present. To identify snoRNA genes, SNOSCAN needs the rRNA (28S, 18S) sequences, whereas a list of rRNA methylation sites is optional. All the hits that had a score higher than 20, a default parameter were considered to be positive candidates. Putative candidates were searched for their location in non-coding and coding regions. Flanking sequences of each snoRNA candidate were examined for any low scoring gene. BLAST was used to find other variants of all snoRNA genes in other *Plasmodium *species [45].

Since *P. falciparum *rRNA methylation sites have not been determined experimentally, an alignment between rRNAs of *S. cerevisiae *and *P. falciparum *was generated, and the *S. cerevisiae *methylated sites were mapped on Pf rRNAs 

### Identification of *Plasmodium *proteins

PlasmoDB and Saccharomyces Genome Database (SGD) databases were searched for genes corresponding to various trans-acting factors involved in the ribosomal biogenesis pathway using BLAST and PSI-BLAST [45].

### Parasite culture

*Plasmodium falciparum *strain 3D7 was cultured in human red blood cells in RPMI 1640 medium supplemented with 50 mg of hypoxanthine/litre, 25 mM NaHCO_3_, 0.2% of glucose, 0.5% of albumax. For most purposes, mixed stage cultures rich in trophozoite stage at high parasitemia (15–20%) were used. To isolate the parasite, the culture was treated with 0.05% saponin to lyse the red blood cell membrane, and the released parasites were pelleted down by centrifugation and washed twice with ice-cold phosphate-buffered saline.

### Experimental verification of snoRNA using northern blot and RT PCR

Total RNA was isolated from the mixed parasite pellet using Trizol Reagent (Invitrogen). cDNA was synthesized using random hexamers as primers, 2 μg of total RNA and SuperScript II reverse transcriptase (RT) (Amersham Pharmacia First strand cDNA sythesis Kit) in a 15 μl reaction mixture. In the control reaction, DNA polymerase was used instead of reverse transcriptase. 1 μl of the cDNA reaction was used to PCR amplify the snoRNA genes using gene specific primers. Northern hybridization: A total of 4–5 μg of total RNA enriched in small RNA (isolated using mirVana miRNA isolation kit from Ambion) was size fractionated on 10% polyacrylamide 8 M-urea gel. Electro-transfer of nucleic acids to nylon charged membrane was carried out in 0.5× Tris-borate-EDTA buffer. Pre-hybridization was carried out for 2 hours in 0.5 M sodium phosphate and 7% SDS buffer. End labeled DNA primers were used as probes for hybridization at 48-degree Celsius. After 20 hour of hybridization, the blots were washed three times in 2× SSC and 0.2% SDS. These blots were exposed to the phosphoimager screen.

### Oligonucleotides

Sequences of oligo-nucleotides used for northern and reverse transcriptase assay are given below. Gene_ S9, Gene_S11 and Gene_14 are the forward primers against the C-terminal end sequence of the protein coding genes PF14_0231, PF14_0230 and PF14_0205 respectively. PFS14_F is the forward primer against PFS14. All other oligo-nucleotides are reverse complementary to the respective snoRNA sequence. Sequences of oligonucleotides are as follows: **PFS1**: AATCAGATTGTGATGCATGAAAA; **PFS2**: TATCAGAAAAATTAATAAAAT AATATGAGC; **PFS3**: TATCAGATTATGCGATTTTCTACA; **PFS4**: TCTCAGTTTTCA AAAATTTCATC; **PFS6**: GATCAGTTGGGCATAGTTATCATCAAAAT; **PFS7**: TATC AGATAGGGATAACTGATTAAAAAT; **PFS8**: TATCATCATAATGCTCTTCAGAC; **P FS9**: GTTCAGATTAATTAATTATCGTCAACAT; **PFS11**: TTTCAGATACTATAATA ATCATC; **PFS12**: TCAGATATAAAATTTATCTTCAC; **PFS13**: TTTCAGTCGTTTTTT TTTCATCTC; **PFS14**: AATCAGAGTAACTATGACTTCTATATAAG; **PFS15**: TTCAG ATAATAGAAGACGAACATA; **PFS16**: AATCAGAAGGTAAAAATATCGAACAT; **P FS17**: GATCAGAACGTTCTACCATCTC; **PFS18**: TTTCAGAGATCTTGGTGAAATA G; **PFS14**_F:AATGTAATAAAAGATTTCTTCTTATATTC; **Gene_S14**: TTATATACA AAAGTTGCATAAATGG; **Gene_S11**: GCTTTATATGTCGAAAAATTACAATAAG; **Gene_S9**: GAAGGAAATATCTGCAAAATTATAAG;

## Authors' contributions

PCM: Design of the study, bioinformatics analysis, experimentation and drafting of the manuscript; AK: participated in reverse transcriptase PCR of PFS14; AS: participated in design of the study, coordination and helped in drafting of the manuscript. All authors have read and approved the manuscript.

## Supplementary Material

Additional file 1 **Proteins involved in ribosomal biogenesis.** This file contains list of all the ribosomal proteins and other protein (PlasmoDB accession number) involved in ribosomal biogenesis in *Plasmodium falciparum*. Click here for file

Additional file 2 **Homologous genes of box C/D snoRNA.** This file contains list of homologue of box C/D snoRNA genes in *P. chabaudi*, *P. berghei*, *P. yoelii*, *P. vivax*, *P. knowlesi *and *P. gallinaceum*. Click here for file

## References

[B1] Snow RW, Guerra CA, Noor AM, Myint HY, Hay SI (2005). The global distribution of clinical episodes of Plasmodium falciparum malaria. Nature.

[B2] Su XZ, Heatwole VM, Wertheimer SP, Guinet F, Herrfeldt JA, Peterson DS, Ravetch JA, Wellems TE (1995). The large diverse gene family var encodes proteins involved in cytoadherence and antigenic variation of Plasmodium falciparum-infected erythrocytes. Cell.

[B3] Gardner MJ, Hall N, Fung E, White O, Berriman M, Hyman RW, Carlton JM, Pain A, Nelson KE, Bowman S, Paulsen IT, James K, Eisen JA, Rutherford K, Salzberg SL, Craig A, Kyes S, Chan MS, Nene V, Shallom SJ, Suh B, Peterson J, Angiuoli S, Pertea M, Allen J, Selengut J, Haft D, Mather MW, Vaidya AB, Martin DM, Fairlamb AH, Fraunholz MJ, Roos DS, Ralph SA, McFadden GI, Cummings LM, Subramanian GM, Mungall C, Venter JC, Carucci DJ, Hoffman SL, Newbold C, Davis RW, Fraser CM, Barrell B (2002). Genome sequence of the human malaria parasite Plasmodium falciparum. Nature.

[B4] Carlton JM, Angiuoli SV, Suh BB, Kooij TW, Pertea M, Silva JC, Ermolaeva MD, Allen JE, Selengut JD, Koo HL, Peterson JD, Pop M, Kosack DS, Shumway MF, Bidwell SL, Shallom SJ, van Aken SE, Riedmuller SB, Feldblyum TV, Cho JK, Quackenbush J, Sedegah M, Shoaibi A, Cummings LM, Florens L, Yates JR, Raine JD, Sinden RE, Harris MA, Cunningham DA, Preiser PR, Bergman LW, Vaidya AB, van Lin LH, Janse CJ, Waters AP, Smith HO, White OR, Salzberg SL, Venter JC, Fraser CM, Hoffman SL, Gardner MJ, Carucci DJ (2002). Genome sequence and comparative analysis of the model rodent malaria parasite Plasmodium yoelii yoelii. Nature.

[B5] Bottger EC (2006). The ribosome as a drug target. Trends Biotechnol.

[B6] Leary DJ, Huang S (2001). Regulation of ribosome biogenesis within the nucleolus. FEBS Lett.

[B7] Smith CM, Steitz JA (1997). Sno storm in the nucleolus: new roles for myriad small RNPs. Cell.

[B8] Kiss T (2002). Small nucleolar RNAs: an abundant group of noncoding RNAs with diverse cellular functions. Cell.

[B9] Mochizuki Y, He J, Kulkarni S, Bessler M, Mason PJ (2004). Mouse dyskerin mutations affect accumulation of telomerase RNA and small nucleolar RNA, telomerase activity, and ribosomal RNA processing. Proc Natl Acad Sci USA.

[B10] King TH, Liu B, McCully RR, Fournier MJ (2003). Ribosome structure and activity are altered in cells lacking snoRNPs that form pseudouridines in the peptidyl transferase center. Mol Cell.

[B11] Gallagher RC, Pils B, Albalwi M, Francke U (2002). Evidence for the role of PWCR1/HBII-85 C/D box small nucleolar RNAs in Prader-Willi syndrome. Am J Hum Genet.

[B12] Kiss T (2001). Small nucleolar RNA-guided post-transcriptional modification of cellular RNAs. Embo J.

[B13] Galardi S, Fatica A, Bachi A, Scaloni A, Presutti C, Bozzoni I (2002). Purified box C/D snoRNPs are able to reproduce site-specific 2'-O-methylation of target RNA in vitro. Mol Cell Biol.

[B14] Decatur WA, Fournier MJ (2003). RNA-guided nucleotide modification of ribosomal and other RNAs. J Biol Chem.

[B15] Lestrade L, Weber MJ (2006). snoRNA-LBME-db, a comprehensive database of human H/ACA and C/D box snoRNAs. Nucleic Acids Res.

[B16] Piekna-Przybylska D, Decatur WA, Fournier MJ (2007). New bioinformatic tools for analysis of nucleotide modifications in eukaryotic rRNA. Rna.

[B17] Vincenti S, De Chiara V, Bozzoni I, Presutti C (2007). The position of yeast snoRNA-coding regions within host introns is essential for their biosynthesis and for efficient splicing of the host pre-mRNA. Rna.

[B18] Fang J, Sullivan M, McCutchan TF (2004). The effects of glucose concentration on the reciprocal regulation of rRNA promoters in Plasmodium falciparum. J Biol Chem.

[B19] van Spaendonk RM, Ramesar J, van Wigcheren A, Eling W, Beetsma AL, van Gemert GJ, Hooghof J, Janse CJ, Waters AP (2001). Functional equivalence of structurally distinct ribosomes in the malaria parasite, Plasmodium berghei. J Biol Chem.

[B20] Lowe TM, Eddy SR (1999). A computational screen for methylation guide snoRNAs in yeast. Science.

[B21] Huttenhofer A, Kiefmann M, Meier-Ewert S, O'Brien J, Lehrach H, Bachellerie JP, Brosius J (2001). RNomics: an experimental approach that identifies 201 candidates for novel, small, non-messenger RNAs in mouse. Embo J.

[B22] Qu LH, Meng Q, Zhou H, Chen YQ (2001). Identification of 10 novel snoRNA gene clusters from Arabidopsis thaliana. Nucleic Acids Res.

[B23] Brown JW, Echeverria M, Qu LH (2003). Plant snoRNAs: functional evolution and new modes of gene expression. Trends Plant Sci.

[B24] Accardo MC, Giordano E, Riccardo S, Digilio FA, Iazzetti G, Calogero RA, Furia M (2004). A computational search for box C/D snoRNA genes in the Drosophila melanogaster genome. Bioinformatics.

[B25] Eo HS, Jo KS, Lee SW, Kim CB, Kim W (2005). A combined approach for locating box H/ACA snoRNAs in the human genome. Mol Cells.

[B26] Luo Y, Li S (2007). Genome-wide analyses of retrogenes derived from the human box H/ACA snoRNAs. Nucleic Acids Res.

[B27] Weber MJ, Luo Y, Li S (2006). Mammalian small nucleolar RNAs are mobile genetic elements. PLoS Genet.

[B28] Venema J, Tollervey D (1999). Ribosome synthesis in Saccharomyces cerevisiae. Annu Rev Genet.

[B29] Mitchell P, Petfalski E, Shevchenko A, Mann M, Tollervey D (1997). The exosome: a conserved eukaryotic RNA processing complex containing multiple 3'-->5' exoribonucleases. Cell.

[B30] Maden BE (1980). Methylation map of Xenopus laevis ribosomal RNA. Nature.

[B31] Escalante AA, Goldman IF, De Rijk P, De Wachter R, Collins WE, Qari SH, Lal AA (1997). Phylogenetic study of the genus Plasmodium based on the secondary structure-based alignment of the small subunit ribosomal RNA. Mol Biochem Parasitol.

[B32] Florens L, Washburn MP, Raine JD, Anthony RM, Grainger M, Haynes JD, Moch JK, Muster N, Sacci JB, Tabb DL, Witney AA, Wolters D, Wu Y, Gardner MJ, Holder AA, Sinden RE, Yates JR, Carucci DJ (2002). A proteomic view of the Plasmodium falciparum life cycle. Nature.

[B33] Gerbasi VR, Weaver CM, Hill S, Friedman DB, Link AJ (2004). Yeast Asc1p and mammalian RACK1 are functionally orthologous core 40S ribosomal proteins that repress gene expression. Mol Cell Biol.

[B34] Chen W, Bucaria J, Band DA, Sutton A, Sternglanz R (2003). Enp1, a yeast protein associated with U3 and U14 snoRNAs, is required for pre-rRNA processing and 40S subunit synthesis. Nucleic Acids Res.

[B35] Brown JW, Echeverria M, Qu LH, Lowe TM, Bachellerie JP, Huttenhofer A, Kastenmayer JP, Green PJ, Shaw P, Marshall DF (2003). Plant snoRNA database. Nucleic Acids Res.

[B36] Piccinelli P, Rosenblad MA, Samuelsson T (2005). Identification and analysis of ribonuclease P and MRP RNA in a broad range of eukaryotes. Nucleic Acids Res.

[B37] Chakrabarti K, Pearson M, Grate L, Sterne-Weiler T, Deans J, Donohue JP, Ares M (2007). Structural RNAs of known and unknown function identified in malaria parasites by comparative genomics and RNA analysis. Rna.

[B38] Huang ZP, Zhou H, He HL, Chen CL, Liang D, Qu LH (2005). Genome-wide analyses of two families of snoRNA genes from Drosophila melanogaster, demonstrating the extensive utilization of introns for coding of snoRNAs. Rna.

[B39] Samarsky DA, Fournier MJ (1999). A comprehensive database for the small nucleolar RNAs from Saccharomyces cerevisiae. Nucleic Acids Res.

[B40] Liang D, Zhou H, Zhang P, Chen YQ, Chen X, Chen CL, Qu LH (2002). A novel gene organization: intronic snoRNA gene clusters from Oryza sativa. Nucleic Acids Res.

[B41] Liang XH, Uliel S, Hury A, Barth S, Doniger T, Unger R, Michaeli S (2005). A genome-wide analysis of C/D and H/ACA-like small nucleolar RNAs in Trypanosoma brucei reveals a trypanosome-specific pattern of rRNA modification. Rna.

[B42] Roy SW, Gilbert W (2006). The evolution of spliceosomal introns: patterns, puzzles and progress. Nat Rev Genet.

[B43] Weischenfeldt J, Lykke-Andersen J, Porse B (2005). Messenger RNA surveillance: neutralizing natural nonsense. Curr Biol.

[B44] Stoeckert CJ, Fischer S, Kissinger JC, Heiges M, Aurrecoechea C, Gajria B, Roos DS (2006). PlasmoDB v5: new looks, new genomes. Trends Parasitol.

[B45] Altschul SF, Madden TL, Schäffer AA, Zhang J, Zhang Z, Miller W, Lipman DJ (1997). Gapped BLAST and PSI-BLAST: a new generation of protein database search programs. Nucleic Acids Res.

