# Supplementary material for: Analysis of small nucleolar RNAs reveals unique genetic features in malaria parasites
Source: BMC Genomics. 2009 Feb 7;10:68. doi: 10.1186/1471-2164-10-68 (PMC2656528; doi:10.1186/1471-2164-10-68)
Supplement: Additional file 1 — Proteins involved in ribosomal biogenesis. This file contains list of all the ribosomal proteins and other protein (PlasmoDB accession number) involved in ribosomal biogenesis in Plasmodium falciparum. [file 1471-2164-10-68-S1.pdf]

Ribosomal proteins:

Large subunit	Plasmodb Acc No.	Small subunit	Plasmodb Acc No.
L3	PF10_0272	SA	PF10_0264
L4	PFE0350c	S2	PF14_0448
L5	PF14_0230	S3	PF14_0627
L6	PF13_0213	S3A	PFC1020c
L7	PFC0300c	S4	PF11_0065
L7A	PF14_0231	S5	PF07_0088
L8	PFE0845c	S6	PF13_0228
L9	PF13_0129	S7	PF13_0014
L10	PF14_0141	S8	PF14_0083
L10A	PF14_0391	S9	PFE1005w
L11	PF07_0079	S10	PF07_0080
L12	PFE0850c	S11	PFC0775w
L13	PF08_0075	S12	PFC0295c
L13A	PF10_0043	S13	PF13_0316
L14	PF14_0296	S14	PFE0810c
L15	PFD0770c	S15	MAL13P1.92
L17	PF13_0268	S15A	PFC0735w
L18	MAL13P1.209	S16	PF08_0076
L18A	PF13_0224	S17	PFL2055w
L19	PFF0700c	S18	PF11_0272
L21	PF14_0240	S19	PFD1055w
L22	PF08_0039	S20	PF10_0038
L23	PF13_0171	S21	PF11_0454
L23A	PF13_0132	S23	PFC0290w
L24	PF13_0049, PFE0300c	S24	PFE0975c
L26	PFC0535w	S25	PF14_0205
L27	PF14_0579	S26	PFB0830w
L27A	PFF0885w	S27	PF13_0045
L28	PF11_0437	S27A	PF14_0027
L29	No Match	S28	PF14_0585
L30	PF10_0187	S29	MAL7P1.300
L31	PFE0185c	S30	PFB0885w
L32	PFI0190w		
L34	PF07_0043		
L35	PF11_0260		
L35A	PF11_0438		
L36	PF11_0106		
L36A	PFC0200w		
L37	MAL7P1.320		
L37A	PFB0455w		
L38	PF11_0312		
L39	PFF0573c		

L40	PF13_0346
L41	PKH_094180
LP0	PF11_0313
LP1	PF11_0043
LP2	PFC0400w
LP3	

U3 snoRNP complex subunit

Subunits	PlasmoDB Acc No.	Description
**IMP3**	PF14_0584	ribosomal protein S4, putative
**IMP4**	PF08_0055	u3 small nucleolar ribonucleoprotein protein
**LCP5**	**No match.**	
**MPP10**	PF10_0266	hypothetical protein
**RRP9**	PFL1290w	U3 snoRNP associated protein, putative
**SOF1**	PFD0455w	ribosomal processing protein, putative

RNA Helicases:

**DBP3**	PFL1310c	ATP-dependent RNA helicase, putative
**DBP4**	PFF1500c	DEAD/DEAH box ATP-dependent RNA helicase, putative
**DBP6**	PFB0860c	RNA helicase, putative
**DBP7**	MAL7P1.113	DEAD box helicase, putative
**DBP8**	PFB0860c	RNA helicase, putative
**DBP9**	PFL2010c	DEAD/DEAH box helicase, putative
**DBP10**	MAL8P1.19	RNA helicase, putative
**DHR1**	PF10_0294, MAL13P1.14	RNA helicase, putative; ATP-dependent DEAD box helicase, putative
**DHR2**	PF10_0294	RNA helicase, putative

**DOB1**	PFF0100w<br>PFI0480w	putative ATP dependent RNA helicase ; helicase with Zn-finger motif, putative
**MTR4**	PFF0100w	Putative ATP dependent RNA helicase
**FAL1**	PF14_0655	RNA helicase-1, putative
**MAK5**	PFE1390w	RNA helicase-1, putative
**ROK1**	PF10_0209	RNA helicase, putative
**RRP3**	PFB0860c	RNA helicase, putative
**SPB4**	PFF1500c	DEAD/DEAH box ATP-dependent RNA helicase, putative
**SEN1**	MAL13P1.13	hypothetical protein, conserved
**DRS1**	PFL2475w	DEAD/DEAH box helicase, putative

Exosome: complex of 3'-5' exonuclease:

**RRP4**	PFD0515w	exosome complex exonuclease rrp4, Putative
**RRP42**	MAL13P1.204	exoribonuclease PH, putative
**CSL4**	MAL7P1.104	3'-5' exoribonuclease Csl4 homolog, putative
**RRP40**	MAL13P1.36	hypothetical protein, conserved
**RRP46**	PF14_0256	exosome complex exonuclease rrp41, putative
**RRP41**	PF14_0256	exosome complex exonuclease rrp41, putative
**RRP44**	MAL13P1.289	mitotic control protein dis3 homologue,putative
**RRP43**	No match	
**RRP45**	PF13_0340	exosome complex exonuclease,

		putative;
**MTR3**	No match	
**RRP6**	PF14_0473	3'-5' exonuclease, putative

5'-3' exonuclease:

**RAT1**	PFI0455w	exoribonuclease, putative
**XRN1**	PFI0455w	exoribonuclease, putative

Nucleo-cytoplasmic Transport factor:

**NOP3**	PF10_0217	pre-mRNA splicing factor,putative
**RNA1**	No match.	
**PRP20**	PFE0420c	guanidine nucleotide exchange factor, putative
**SRM1**	PFE0420c	guanidine nucleotide exchange factor, putative
**SRP1**	PF08_0087	importin alpha, putative

Nucleolar protein:

**DIM1**	PF14_0156	dimethyladenosine transferase, putative
**NSR1**	PF14_0194	spliceosome-associated protein
**NOP2**	PF11_0305	
**NOP4**	PFI0820c	RNA-binding protein, putative
**NOP8**	PF11_0239	protein kinase, FIKK family

**NPI46**	PFL2275c	FK506-binding protein (FKBP)-type peptidyl-propyl isomerase
**RRP8**	PFI1235w	methyltransferase, putative
**RRP5**	PF14_0042	U3 small nucleolar ribonucleoprotein, U3 snoRNP, putative

Box H/ACA snoRNP complex:

**NHP2**	PFD0960c	ribosomal protein L7Ae-related protein, putative
**CBF5**	PF14_0174	Small nucleolar ribonucleoprotein, snoRNP, putative
**GAR1**	PF13_0051	snornp protein gar1 homologue, putative
**NOP10**	PF14_0784	Ribosome biogenesis protein, NOP10-like

Box C/D snoRNP complex:

**NOP1**	PF14_0068	fibrillarin, putative
**SNU13**	PF11_0250	high mobility group-like protein NHP2, putative
**NOP56**	PF11_0191	nucleolar protein NOP56, putative
**NOP58**	PF10_0085	nucleolar protein NOP5, putative

RNase P/MRP complex subunits:

**POP1**	MAL7P1.28	ribonucleases p/mrp

		protein subunit, putative
**POP3**	No match	
**POP4**	PFF1355w	Hypothetical protein
**POP5**	No match	
**POP6**	No match	
**POP7**	PFB0735c	Hypothetical protein
**SNM1**	No match	
**RMP1**	No match	
**POP8**	No match	
**RPP1**	PFF1040w	Hypothetical protein
