# Supplementary material for: Analysis of small nucleolar RNAs reveals unique genetic features in malaria parasites
Source: BMC Genomics. 2009 Feb 7;10:68. doi: 10.1186/1471-2164-10-68 (PMC2656528; doi:10.1186/1471-2164-10-68)
Supplement: Additional file 2 — Homologous genes of box C/D snoRNA. This file contains list of homologue of box C/D snoRNA genes in P. chabaudi, P. berghei, P. yoelii, P. vivax, P. knowlesi and P. gallinaceum. [file 1471-2164-10-68-S2.pdf]

Additional file 2: Homologs of snoRNA in *Plasmodium* species

snoRNA	PC Ac no.	Start	End	PY Ac no.	Start	End	PV Ac no.	Start	End
**S1**	PC_RP1947	560	484	MALPY01185	16	93	ctg_7187	314148	314070
**S2**	PC_RP2105	10261	10178	MALPY00145	12	98	ctg_6950	235021	235098
**S3**	PC_RP4763	270	350	N.A.	N.A.	N.A.	ctg_6977	277923	277842
**S4**	N.A.	N.A.	N.A.	N.A.	N.A.	N.A.	ctg_6977	292625	292545
**S5**	PC_RP1727	2540	2473	MALPY00191	4647	4582	ctg_6977	288677	288611
**S6**	PC_RP1727	1097	1034	MALPY00191	3090	3027	ctg_6977	286891	286826
**S7**	PC_RP1727	1859	1776	MALPY00191	3902	3818	ctg_6977	287754	287674
**S8**	PC_RP4842	16	95	MALPY02383	9609	9530	ctg_6877	1056444	1056360
**S9**	PC_RP0299	677	761	MALPY02194	8235	8313	ctg_6877	1058943	1058874
**S9b**	PC_RP0299	725	793	MALPY02194	8277	8345	N.A.	N.A.	N.A.
**S10**	PC_RP1988	1126	1062	MALPY02194	3506	3442	ctg_6877	1064434	1064496
**S11**	PC_RP1988	363	291	MALPY02194	2651	2579	ctg_6877	1065169	1065246
**S12**	PC_RP1988	105	28	MALPY02194	2386	2309	ctg_6877	1065653	1065731
**S13**	PC_RP4842	634	709	MALPY02383	9009	8934	ctg_6877	1055723	1055648
**S14**	PC_RP2302	226	355	MALPY01189	901	769	ctg_6877	1161717	1161809
**S15**	N.A.	N.A.	N.A.	MALPY00036	23046	22972	ctg_6877	1993544	1993622
**S16**	N.S	N.S	N.S	N.S	N.S.	N.S	N.S	N.S	N.S
**S17**	PC_PH5126	1009	1083	MALPY00366	5511	5437	ctg_6871	467845	467917
**S18**	PC_RP1995	5074	5003	MALPY00606	10035	9964	ctg_6871	128896	128964

snoRNA	PK Ac no	Start	End	PB Ac no	Start	End	PG Ac no	Start	End
**S1**	PKN.002526	6338	6416	PB_RP2870	12315	12238	Pg_2570384.c000316052. Contig1	6692	6768
**S2**	PKN.002365	125834	125916	PB_RP2232	3657	3740	Pg_2570384.c000319723. Contig1	2078	1999
**S3**	PKN.002740	45270	45191	PB_RP2363	860	780	N.A.	N.A.	N.A.
**S4**	PKN.002161	1459	1379	PB_RP2474	2419	2337	N.A.	N.A.	N.A.
**S5**	PKN.002740	55644	55577	PB_PH3411	758	825	Pg_2570384.c000013797. Contig1	2075	2007
**S6**	PKN.002740	53816	53751	N.A.	N.A.	N.A.	Pg_2570384.c000013797. Contig1	404	339
**S7**	PKN.002740	54712	54631	NA	N.A.	N.A.	Pg_2570384.c000013797. Contig1	1343	1263
**S8**	PKN.002755	78400	78316	PB_RP1551	4237	4158	N.A.	N.A.	N.A.
**S9**	PKN.002755	81067	80995	PB_RP1105	795	727	N.A.	N.A.	N.A.
**S9b**	N.A.	N.A.	N.A.	PB_RP1105	839	759	Pg_2570384.c000014017. Contig1	970	902
**S10**	PKN.002755	87058	87121	PB_RP3524	2515	2451	N.A.	N.A.	N.A.
**S11**	**PKN.002755**	**87918**	**88001**	PB_RP3524	1715	1643	N.A.	N.A.	N.A.
	**PKN.000135**	**8511**	**8594**	N.A.	N.A.	N.A.	N.A.	N.A.	N.A.
**S12**	PKN.002755	88508	88586	PB_RP3524	1457	1381	N.A	N.A.	N.A.
**S13**	PKN.002755	77587	77512	PB_RP1551	3593	3518	N.A.	N.A.	N.A.
**S14**	PKN.002755	183962	184106	PB_RP3214	4921	5052	N.A.	N.A.	N.A.
**S15**	PKN.002765	490292	490369	PB_RP2926	5983	5909	N.A.	N.A.	N.A.
**S16**	N.S	N.S	N.S	N.S	N.S	N.S	N.S	N.S	N.S
**S17**	PKN.002763	189342	189414	PB_RP0435	1462	1388	Pg_2570384.c000320327. Contig1	26	96
**S18**	PKN.002488	29130	29061	PB_RP2768	3268	3197	N.A.	N.A.	N.A.

List of homologues of box C/D snoRNA genes (PlasmoDB Accession number) in other *Plasmodium* species: *P. chabaudi* (PC), *P. yoelii* (PY), *P. vivax* (PV), *P. knowlesi* (PK) and *P. gallinaceum* (PG). “NS” means snoRNA was absent at orthologous loci whereas “NA” stands for those snoRNA which are located in the regions lacking any sequencing information. PFS11 has two homologs in PK, which are highlighted in bold.
